# Breathing Pattern Interpretation as an Alternative and Effective Voice Communication Solution

**DOI:** 10.3390/bios8020048

**Published:** 2018-05-15

**Authors:** Yasmin Elsahar, Kaddour Bouazza-Marouf, David Kerr, Atul Gaur, Vipul Kaushik, Sijung Hu

**Affiliations:** 1 Wolfson School of Mechanical, Electrical, and Manufacturing Engineering, Loughborough University, Loughborough, Leicestershire LE11 3TU, UK; Y.Elsahar@lboro.ac.uk (Y.E.); k.bouazza-marouf@lboro.ac.uk (K.B.-M.); D.Kerr@lboro.ac.uk (D.K.); 2 University Hospitals of Leicester NHS Trust, Leicester, LE3 9QP, UK; gaur_atul@hotmail.com (A.G.); vipul.kaushik@uhl-tr.nhs.uk (V.K.)

**Keywords:** breathing interpretation, alternative and augmentative communication, acoustic sensors, pattern classification, voice communication

## Abstract

Augmentative and alternative communication (AAC) systems tend to rely on the interpretation of purposeful gestures for interaction. Existing AAC methods could be cumbersome and limit the solutions in terms of versatility. The study aims to interpret breathing patterns (BPs) to converse with the outside world by means of a unidirectional microphone and researches breathing-pattern interpretation (BPI) to encode messages in an interactive manner with minimal training. We present BP processing work with (1) output synthesized machine-spoken words (SMSW) along with single-channel Weiner filtering (WF) for signal de-noising, and (2) *k*-nearest neighbor (*k-NN*) classification of BPs associated with embedded dynamic time warping (DTW). An approved protocol to collect analogue modulated BP sets belonging to 4 distinct classes with 10 training BPs per class and 5 live BPs per class was implemented with 23 healthy subjects. An 86% accuracy of *k-NN* classification was obtained with decreasing error rates of 17%, 14%, and 11% for the live classifications of classes 2, 3, and 4, respectively. The results express a systematic reliability of 89% with increased familiarity. The outcomes from the current AAC setup recommend a durable engineering solution directly beneficial to the sufferers.

## 1. Introduction

The escalation in the numbers of speech-disabled individuals renders a rising need for augmentative and alternative communication (AAC) solutions [1,2]. AAC techniques range in complexity and are classified into three main categories: *no-technology* AAC (interpretation of gestures and body movements), *low-technology* AAC (basic communicative aids, books, and board displays), and *high-technology* AAC (software designed to work with a machine) [3]. Existent interdisciplinary AAC methods rely on machine interpretation of sets of purposeful gestures and movements. Such procedures are beneficial yet require significant efforts from users in terms of gesture acquisition and practice. Moreover, the predefinition of word sets offered by most solutions confines the conveyance of user-specific messages. AAC researchers confirm that there is still wide room for technology utilization and improvement in system versatility to train and adjust the devices [4,5].

Breathing consists of inhalation followed by exhalation, separated by a short pause. In medical terminology, the term respiratory rate is the measurement of breathing cycles per minute [6]. Clinical observations express typical respiratory frequency ranges of 0.2–0.8 Hz [7]. The detection of human breathing can be accomplished through different modalities that expand to comprise a wide range of techniques. Common collection methods include the usage of electroencephalogram (EEG) signals [8], smart wearable garments with fiber optic sensors [9], photoplethysmogram measurements of cardiac activity [10], pressure and thermal sensors [11], and airflow examination [8]. The utilization of acoustic signals and microphones to capture breathing signals is common; however this is for clinical respiratory measurement purposes [8,12].

Limited research has been carried out for the translation of breathing signals into synthesized machine-spoken words (SMSW). Early studies have started with the usage of sniffing signals in the scope of AAC and in the control of devices for paralysis sufferers and individuals in locked-in syndrome. The fine control of sniffing is measured through nasal pressure and has been utilized to write text or drive a wheelchair [13]. Fine breath tuning has also aided communicative needs of AAC users through Dasher, a text-entry tool available on several operating systems. It uses input from a mouse (or other means) to manipulate a cursor on a screen to steer towards alphabetical letters and start writing. A predictive language model accompaniment displays probable words for the facilitation of the writing process. In the breath-triggered mode, AAC users navigate Dasher’s software panels using a thoracic belt worn around the chest. The belt expands and contracts with breathing movements, guiding the cursor of a special mouse towards cascading letters in the system. With increased familiarity with this platform, well-trained users were reported to write English letters at a rate of 15 words per minute [14]. However, the inhalation and exhalation movements used for the expansion and contraction of the belt could be restrictive. Instead, breathing variations could be detected through pressure or airflow sensors. A recent invention, “TALK” correspondingly uses distinct inhalation and exhalation breathing signals together with a low-cost micro-controller board to encode messages through Morse codes [4]. Generally, the main limitation of the digital encoded representations of breathing signals in the above-listed studies is in the restricted direct information content of the signals. Variations in breathing amplitudes, phases, and specific personal traits could be representative of unique and comprehensive messages to be directly used for communicative purposes [4]. Moreover, the need to input letters to form words infers slow conversational rates. Under the analogue scheme, a study used a medical breathing mask together with processing electronics and software to interpret patterns of breathing through pressure variations [4]. The approach showed primary success in terms of utilizing breathing for the purpose of AAC. Analogue encoding provides increased bandwidth at the low breathing frequencies by utilizing amplitude, frequency, and phase changes to encode users’ intended meanings.

The aim of this study is to research breathing pattern interpretation (BPI) as an alternative yet effective AAC solution by using the modulation of acoustic breathing patterns (BPs) to output synthesized-voice-format messages. Users breathe effortlessly and learn to generate modulated patterns picked up by a microphone to be translated to their choice of machine-spoken words. Simultaneously, the system learns to recognize BPs along with the associated words to SMSW whenever a BP is triggered. The communication process becomes unbound to pronunciation or gestural movements that may be cumbersome to learn. Several algorithms exist in the literature for classifying time series data [15,16]. Reliable recognition and high-quality signals are the basic elements for successful classification [17]. The study looks at the determination of a suitable pattern classification approach for BP recognition. Filtering and standard de-noising techniques are adapted to enhance breath signal quality and to increase the total signal-to-noise ratio (SNR) [18].

## 2. Materials and Method

### 2.1. Overview of BPI Operational Modes

**Figure 1:**
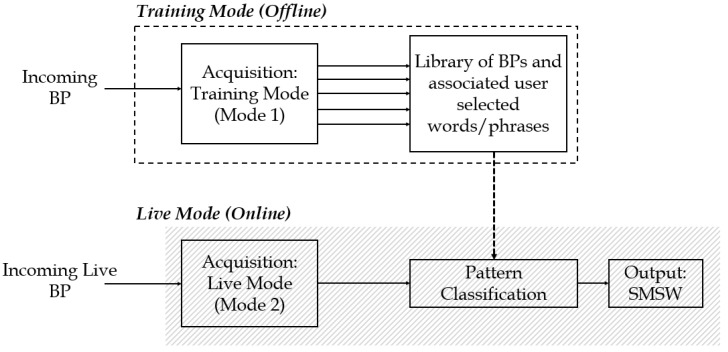
Overview of the two modes of operation: training operational mode and live operational mode.

Figure 1 demonstrates the two operational modes supported by the system. The *Offline* mode consists of the acquisition of training BP patterns. This expands to include BP libraries and user-intended vocabulary linked with each BP. The *Online* mode consists of live pattern acquisition for classification by the machine to output SMSW. Potential system users should possess the ability of breathing modulation and a level of cognitive ability that enables them to operate the device.

### 2.2. BPI System Architecture

**Figure 2:**
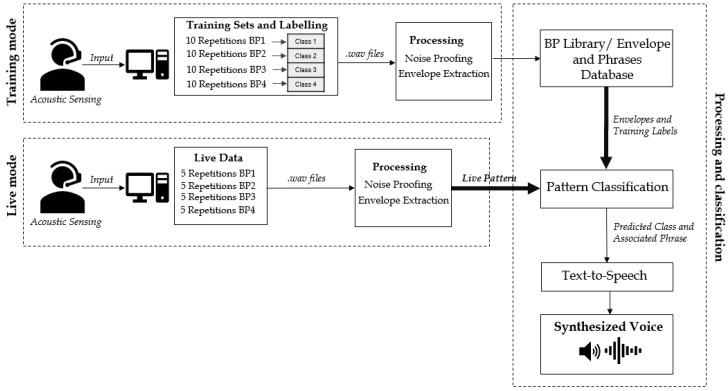
Proposed breath-to-speech system architecture, including the training mode, live mode, processing, and pattern classification.

The proposed system architecture is presented in Figure 2. BP classification and word synthesis are the core elements of the implementation. In Figure 2, a sample of four distinct sets of training and live signals is depicted.

### 2.3. Experimental Protocol

**Figure 3:**
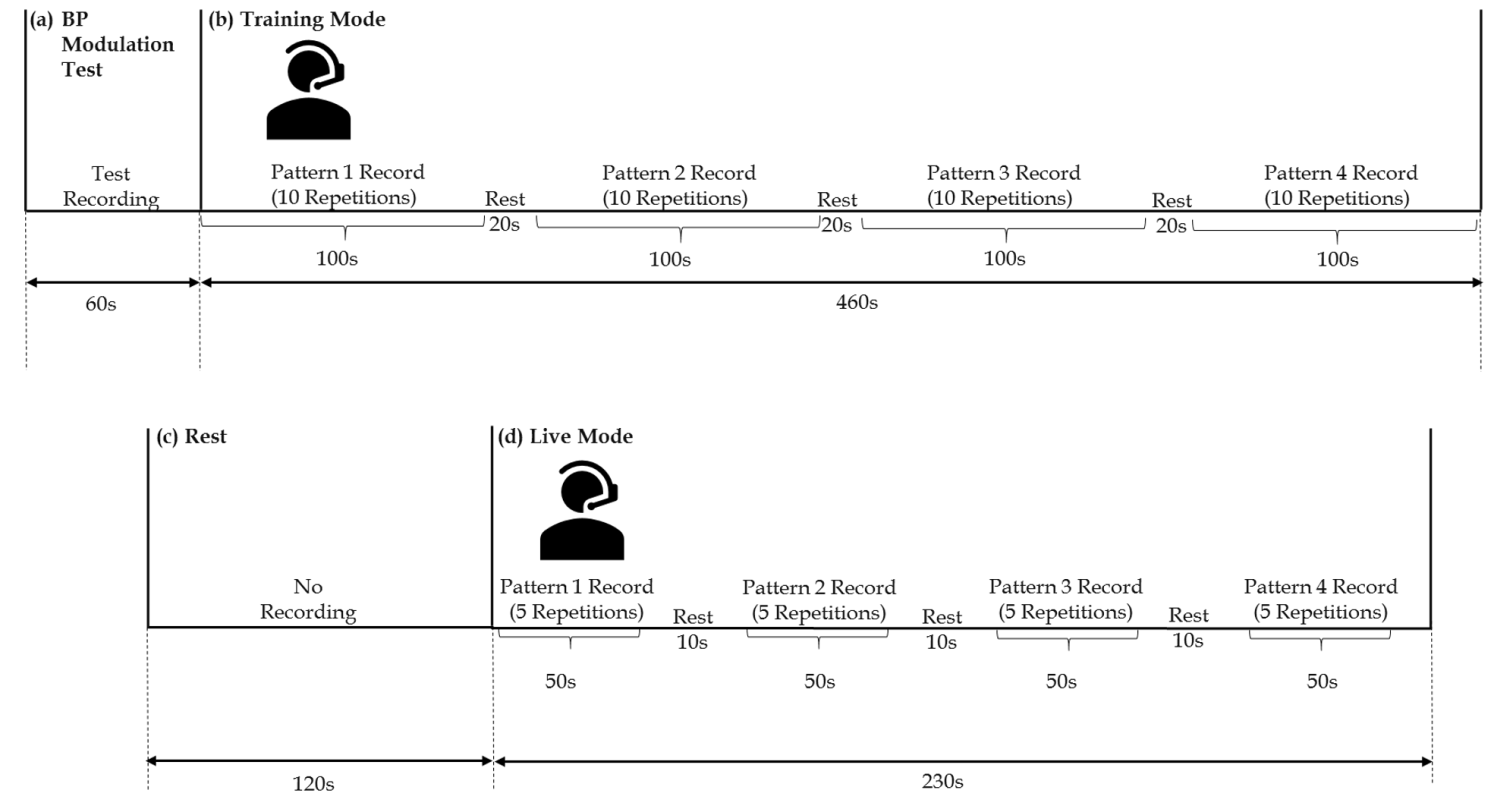
Experimental setup of acoustic breathing pattern (BP) detection, presented with the subject using the head-band unidirectional microphone (UM). The allocated durations are shown for (**a**) modulation testing, (**b**) BP acquisition in the training mode, (**c**) the rest duration between the recorded sets, and (**d**) BP acquisition in the live mode.

The experimental protocols were implemented with the approval of Loughborough University’s Ethics Committee. Informed consent was obtained from all subjects prior to participation. The protocol was implemented with the participation of 23 healthy subjects sitting in an environment-controlled room (16–18 ${}^{\circ }$C, Relative Humidity (RH): 50–70%). An ultralight 7 g head-band cardioid unidirectional microphone (UM) (Monacor Stageline HSE-152/SK, back electret, sensitivity of 1.8 mV/Pa at 1 kHz) connected to a 64-bit operating system running MATLAB (Version 2016a) was used. For hygiene purposes, replacement of the microphone foam covers was carried out following each subject’s session. The subjects were guided through the process of recording modulated BPs and testing the platform prior to the start of the recording. Each subject made a selection of four BPs of his/her choice. As shown in Figure 3, each BP was recorded over a 10 s window, and 10 repetitions were recorded for each of the 4 BP classes to represent the training set (total: 40 training BPs). Further guidance throughout the process of BP modulation was provided during the acquisition of the first class of training BPs. The subjects were allowed 20 s of rest between the acquisition of training classes and a 2 min rest after the completion of the training mode. Five extra “live” repetitions of each of the four BP classes were acquired to be used as the test sets. Ten seconds of rest was provided between the acquired live sets.

### 2.4. BP Processing

**Figure 4:**
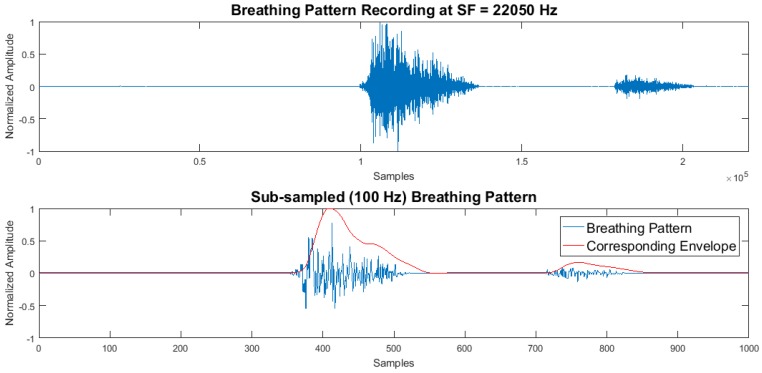
Subsampling of acoustic breathing patterns, with a sampling frequency of 22,050 Hz (**top**) and 100 Hz (**bottom**).

The sound acquisition and recordings were completed using MATLAB. BPs were recorded from the subjects at a rate of 22,050 Hz and an initial silence duration of 1 s. The signals were filtered to suppress the contaminating ambient noise resulting from sources in the background or poor signal quantization using the Weiner filtering (WF) approach in [19,20] following the assumption that noise is stationary over the acquired time window. The decision-directed method for noise reduction in [19] tracks the a priori SNR through the computation of the a posteriori SNR on the basis of the assumption that *SNR*_*a*-posteriori_ = *SNR*_*a*-priori_ + 1. The a posteriori SNR was calculated from the corrupted signal and the noise variance. The a priori SNR was tracked through the decision-directed method in [21], and the Weiner estimate gain function was found using the a priori SNR through

(1) $$W\left(f\right)=\frac{\mathrm{SNR}(f)}{\mathrm{SNR}(f)+1},$$

where *f* is the discrete frequency variable [19,20]. Breathing signals were sub-sampled to 1000 Hz for dimension reduction, with no violation to anti-aliasing. BP envelopes were extracted using a Butterworth low-pass filter with a cut-off frequency of 1/(0.25×SF). The negative portions of the signals were discarded, as proposed by [12]. The attained BP envelopes’ representative of inhalation and exhalation were further sub-sampled to 100 Hz per pattern to speed up computations. The information content was minimized to reduce time complexities; however, the instances of exhalation and inhalation were identifiable, as displayed in Figure 4.

**Figure 5:**
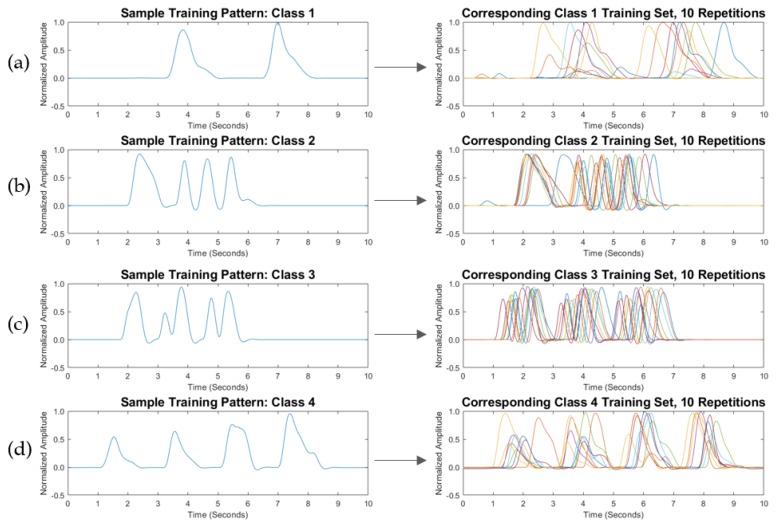
Training breathing pattern (BP) set (user-selected), with (**a**) sample of class 1 BP pattern, followed by 10 BP repetitions of class 1; (**b**) sample of class 2 BP pattern, followed by 10 BP repetitions of class 2; (**c**) sample of class 3 BP pattern, followed by 10 BP repetitions of class 3; and (**d**) sample of class 4 BP pattern, followed by 10 BP repetitions of class 4.

**Figure 6:**
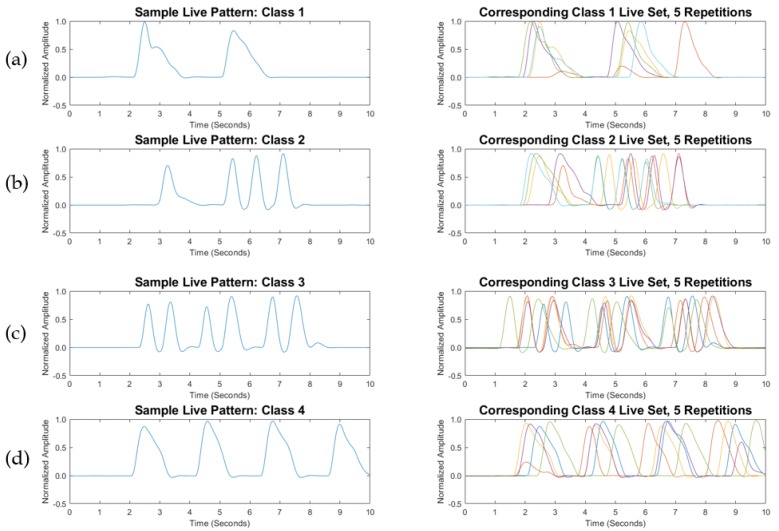
Associated live breathing pattern (BP) set for the training set displayed in Figure 5, with (**a**) sample of class 1 live BP pattern, followed by five live BP repetitions of class 1; (**b**) sample of class 2 live BP pattern, followed by five live BP repetitions of class 2; (**c**) sample of class 3 live BP pattern, followed by five live BP repetitions of class 3; and (**d**) sample of class 4 live BP pattern, followed by five live BP repetitions of class 4.

Each training BP envelope was assigned to a class and a label that was passed to the classifier. Training BPs were labeled as patterns 1–4. Figure 5 presents a sample training set, and Figure 6 presents the corresponding live set. In Figure 5 and Figure 6, a sample BP envelope is displayed for each of the training and the live classes, followed by the corresponding repetitions of the training set (10 repetitions per class) and the live set (5 repetitions per class). Every class was assigned to a specific word or phrase. In practice, words and phrases are user defined and can be changed according to an individual’s needs. An example of a training library for four BPs is shown in Table 1. The training BP envelopes were saved in a separate data file for classification against the acquired live inputs.

### 2.5. BP Classification and SMSW

**Figure 7:**
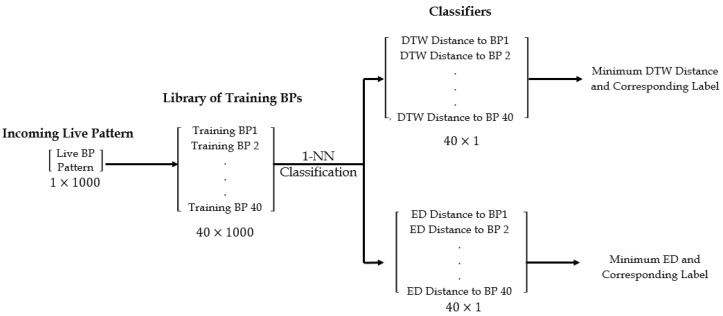
Classification operations, showing the arrangements of the live breathing pattern (BP) matrix, the training BP matrix, and the associated classification distances.

Classic Euclidean distance (ED) and dynamic time warping (DTW) were embedded in a *k*-nearest neighbor (*k*-NN) classifier, with k=1. The ED is given by

(2) $$d\left(U,V\right)=\left(\sum_{i\;=\;1}^{m}{\left(u_{i}-v_{i}\right)}^{2}\right){}^{1/2},$$

where *u_i_* and *v_i_* are the *i*th features of time series *U* and *V*, respectively. On the other hand, DTW uses dynamic programming to search for flexible similarity measures between temporal series. For two time series *U* and *V* of lengths *a* and *b*, respectively, a warp path *W* = *w*_1_, … , *w_K_* can be constructed such that $w_{k}={\left(i,j\right)}_{k}$, where *i* and *j* are indexes from time series *U* and *V*, respectively, and max(*b*,*a*) ≤ K < b+a−1. Constraints associated with the warping path include conforming to boundary conditions, with *W*_1_ = (1,1) and *W*_K_ = (*a*,*b*); continuity; and monotonicity. The distance path minimizing the cost between the series can be found through

(3) $$D\left(i,j\right)=d\left(u_{i},v_{j}\right)+min\left\{\begin{array}{c} D(i,j-1)\\ D(i-1,j)\\ D(i-1,j-1)\; \end{array}\right.$$

where $d(u_{i},v_{j})$ is the distance between $u_{i}$ and $v_{j}$ [22]. In this study, $d(u_{i},v_{j})$ was computed using the ED. A one-nearest neighbor *1-NN* classification coupled with a warped distance is hypothesized as a powerful candidate in time series classification [22,23]. DTW has an $O(N^{2})$ time complexity [24]. On the other hand, the ED is assumed to be common, faster, and less computationally demanding. BPs were normalized and the offset was removed. In MATLAB, training BPs for the four classes were arranged in a 40 by [SF×10] matrix. A function was created to measure the DTW distance between the training patterns and the live input. The output of the defined function was an [M×1] vector containing computed DTW distances between the live sample’s envelope and the first to *M*th training BP envelopes. The ED was directly embedded in the 1-NN classifier, and the distance to the *M*th training BP envelope was similarly computed. Classification operations are displayed in Figure 7. The word/phrase corresponding to the label of the smallest distance and the BP matching envelope were assigned to a new string variable passed to the machine’s default synthesizer through MATLAB to output SMSW.

## 3. Results

BP classification results were analyzed for each separate class. For 23 subjects, 92 *sets* of live modulated BPs were acquired for the four classes, with a total of 460 live BP signals. An individual confusion matrix (CM) was created for every subject on the basis of the ED and DTW 1-NN classification. The predicted and actual class labels were used for the creation of a percentage value representative of the correct classifications per subject. This was attained from the ratio of correct live BP classifications to the total of the 20 live BPs. The results were aggregated for all 23 subjects who participated in the experimental protocol. Seven sets of outliers were omitted for subjects who had difficulties recalling the selected BP.

**Figure 8:**
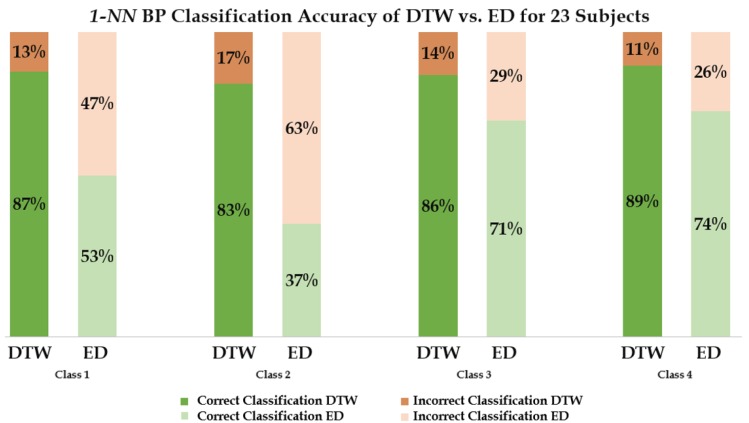
Four classes’ classification performance with dynamic time warping (DTW) and Euclidean distance (ED).

The classification accuracy for a 1-NN classifier using both the embedded DTW measure and the default ED are shown in Table 2 and Table 3. These two tables display the CMs pertaining to the four classes of the BPs and the percentage of correct and incorrect classifications among the 23 subjects for each distance measure of each class. Figure 8 gives a comparative bar chart, which contrasts with the highest classification success rates belonging to the two techniques (ED and DTW). The highest classification success rates for each technique as shown in Table 2 and Table 3 are appropriately colored with respect to the bar graph in Figure 8. A reliability rate of 89% was found with DTW classified BPs in comparison to 74% using the ED with increased user familiarity with the platform. Average rates are also indicative of an overall performance of 86% for the DTW BP classification (error rate of 14%) in comparison to 59% for the ED BP classification (error rate of 41%).

The examined data show that the performance of the ED was less reliable as the BPs became more structurally complex. The BP sequences suffered mismatches related to speeds of pattern repetition and temporal shifts, even over the short examined window of 10 s. DTW-classified series were more robust to temporal mismatches, however, at the cost of a heavier computational complexity. Hence, sub-sampling of the BP time series was rendered essential. Following the BP classification, the synthesized words/phrases were spoken on the basis of matching the label of the selected training envelope with the associated phrase. A correct classification guaranteed the immediate production of the desired word/phrase.

## 4. Discussion

During the BP acquisition phases, the utilization of the cardioid UM aided in the limitation of audible noise collection by the microphone. Moreover, filtering stationary background noises through the single-channel Weiner filter ensured better signal quality. Minimal variations in the acquisition process resulted from the dependability of angles of recording on subjects’ preferred UM positioning in relation to the UM’s diaphragm. Breathing intensity at the input of the microphone was hence slightly variant among the subjects [8]. However, variations were minimal between the training and live datasets of the same subject, with limited effects on classification accuracy.

For BP classifications, an increase in user familiarity with the platform was predominant with patterns belonging to the fourth class. In class 1, necessary guidance regarding BP modulation was provided to the subjects during the acquisition. This resulted in a cumulative error rate of 13.0% with 1-NN DTW. There was no guidance provided for subsequent classes. Generally, as the subjects became more acquainted with the platform, the process of BP modulation was easier to attain. In turn, any experienced users were found to have a better control of the AAC device. DTW classification of the BPs of the final class (class 4) showed both the least number of omitted patterns and the highest rate of classification successes, as per Table 2. The error rates for 1-NN DTW decreased by 17%, 14%, and 11% for the live classifications of classes 2, 3, and 4, respectively.

In light of the obtained data, the variations in speeds and data shifts of the modulated BPs were seen to be inevitable, as the live patterns did not possess the exact compositions of the training patterns; 1-NN ED deteriorated with increasing numbers of BPs, even over short time windows. For instance, by examining the third class of the sample BP corresponding to the training and live sets in Figure 5 and Figure 6, the live BP set was shown to be more stretched over the allocated window. In consequence, 1-NN ED did not correctly classify this live BP set, while 1-NN DTW correctly classified the entire set. Although DTW is a “loose” metric, previous empirical research on large sets of speech signals shows that DTW could be used to classify audio signals [25]. Moreover, the warping complexity was managed through the restriction of each BP in this study to a sampling frequency of 100 Hz over the short recording time window.

The implementation of fast DTW for real-time applications will be addressed in the forthcoming developments of the system. Moreover, in the current prototype system, the user is prompted to input the BP by pressing on the “Enter” button on a keyboard. However, this would not be an ideal solution in a real system. Therefore, automatic recognition of the modulated BPs together with the recognition of more than four user-defined BPs will need to be addressed. Preliminary tests have been carried out using machine learning techniques with deep learning (DL) functionality. This is being investigated to evaluate its robustness with different users and will be reported in a future study.

## 5. Conclusions

The modulation of BPs and translation into SMSW are presented in this study. The processing and classification of acoustically collected breathing signals along with associated BPI assumptions are described for the development of the proposed AAC solution. The endorsed BP training and live modes for AAC communication are detailed in Figure 1. The collection and proofing of the BPs were attained through the utilization of the UM together with single-microphone noise filtering. The BP envelope extraction along with training set creation and associated vocabulary were accomplished, with four distinct classes of BPs.

BP classification accuracies were obtained through cumulative CMs of 23 subjects, with 1-NN classifiers embedded with ED and DTW measures. Temporal variations in the BP sets confined the usability of 1-NN ED to BP classification. The experimental results for BP classification through 1-NN DTW at a rate of 100 Hz showed a reliability of 89% with increased familiarity with the AAC system. The outcome from the present study reveals a durable and conceptual solution to meet the growing demand of one of today’s AAC areas related to synthesized speech production. A one-stop engineering approach can be recommended as a future expansion of the study by embedding software and hardware to attain an all-in-one plug-in solution.

## Figures and Tables

**Table 1:** Sample breathing pattern (BP) to text vocabulary. The label of every BP of the four acquired training classes is mapped to a phrase.

Class	Label	Transit Language
1	Breath_Pattern_1	“Hello, good morning”
2	Breath_Pattern_2	“Thank you”
3	Breath_Pattern_3	“My name is …”
4	Breath_Pattern_4	”May I have a train ticket please?”

**Table 2:**
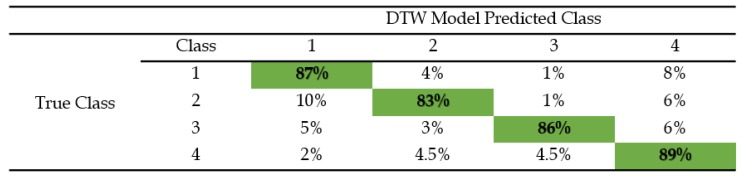
Confusion matrix of *1-NN* DTW, showing cumulative classification accuracy of the predicted BPs classes in comparison with the true BP classes. The highest classification rates are given in dark green.

**Table 3:**
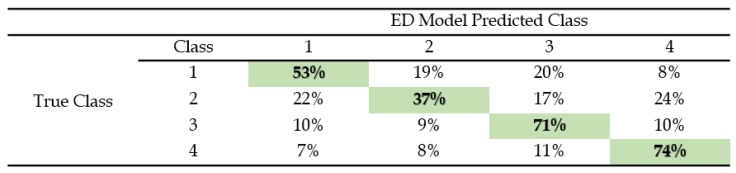
Confusion matrix of *1-NN* ED, showing cumulative classification accuracy of the predicted BPs classes in comparison with the true BP classes. The highest classification rates are given in light green.
